# Post-traumatic Stress Symptoms and Post-traumatic Growth in 223 Childhood Cancer Survivors: Predictive Risk Factors

**DOI:** 10.3389/fpsyg.2016.00287

**Published:** 2016-02-29

**Authors:** Marta Tremolada, Sabrina Bonichini, Giuseppe Basso, Marta Pillon

**Affiliations:** ^1^Department of Developmental and Social Psychology, University of PaduaPadova, Italy; ^2^Department of Child and Woman’s Health, Oncology Hematology Division, University Hospital of PaduaPadova, Italy

**Keywords:** post-traumatic stress symptoms, post-traumatic growth, cancer, pediatric, survivors, development, risk predictors, perceived social support

## Abstract

With modern therapies and supportive care, survival rates of childhood cancer have increased considerably. However, there are long-term psychological sequelae of these treatments that may not manifest until pediatric survivors are into adulthood. The prevalence of post-traumatic stress disorder in young adult survivors of childhood cancer ranges from 6.2 to 22%; associated risk factors are young age at the assessment, female gender, low education level, and some disease-related factors. The aim of this study was to investigate, in adolescent and young adult (AYA) survivors of childhood cancer, the incidence and severity of post-traumatic stress symptoms (PTSSs), and to identify the risk factors and the associated post-traumatic growth (PTG) index. Participants were 223 AYA cancer survivors recruited during follow-up visits in the Oncohematology Clinic of the Department of Child and Woman’s Health, University of Padua. Data were collected from self-report questionnaires on PTSS incidence, PTG mean score, perceived social support, and medical and socio-demographic factors. Ex-patients’ mean age at the assessment was 19.33 years (*SD* = 3.01, 15–25), 123 males and 100 females, with a mean of years off-therapy of 9.64 (*SD* = 4.17). Most (52.5%) had survived an hematological disorder and 47.5% a solid tumor when they were aged, on average, 8.02 years (*SD* = 4.40). The main results indicated a moderate presence of clinical (≥9 symptoms: 9.4%) and sub-clinical PTSS (6–8 symptoms: 11.2%), with the avoidance criterion most often encountered. Re-experience symptoms and PTG mean score were significantly associated (*r* = 0.24; *p* = 0.0001). A hierarchical regression model (*R*^2^ = 0.08; *F* = 1.46; *p* = 0.05) identified female gender (β = 0.16; *p* = 0.05) and less perceived social support (β = -0.43; *p* = 0.05) as risk factors to developing PTSS. Another hierarchical regression model assessed the possible predictors of the PTG total score (*R*^2^ = 0.36; *F* = 9.1; *p* = 0.0001), with female gender (β = 0.13; *p* = 0.04), actual age (β = 0.52; *p* = 0.0001), younger age at the diagnosis (β = -0.3; *p* = 0.02), and less years off-therapy (β = -0.58; *p* = 0.0001) impacting on PTG.

## Introduction

### Psychological Late Effects in Pediatric Cancer Survivors

The experience of treatment for pediatric cancer is an exhausting, time- and energy-consuming, complex process (Sloper, 2000) that may change children’s development, personality and relationships with their family and peers. Although, recent advances in treatment have led to a significant increase in survival (Homer et al., 2009), children often undergo multimodal treatment including surgery, chemotherapy and radiation, which can cause numerous long-term side effects, both physical and psychological (Pui et al., 2003). In this study we consider global psychological well-being, focusing on two important aspects: post-traumatic stress symptomatology (PTSS) and post-traumatic growth (PTG).

Recognition and utilization of the concepts of PTSS and PTG in childhood cancer survivors bestow a number of advantages; psychological reactions and possible resilience factors can thereby be recognized in order to provide psychotherapeutic and specific interventions for those ex-patients who need them.

Patenaude and Kupst (2005) reviewed all the studies dealing with psycho-social functioning in pediatric cancer, emphasizing that no child with cancer remains unchanged by the experience. Most studies have found little evidence of serious maladjustment or maladaptation in pediatric cancer patients: most survivors showed good adjustment on psychological self-report measures and their scores were not significantly different from those of the norms, controls or comparison groups. Similarly, they tend to have fewer emotional and behavioral problems, based on reports of others (e.g., teachers, parents, peers; Patenaude and Kupst, 2005). Other studies (Eiser et al., 2000; Zebrack and Zeltzer, 2003) found that survivors did not show deficits in measures of anxiety, depression, or self-esteem when compared to the general population. However, these findings must be viewed with caution. Some researchers have suggested that comparing symptomatology scores of children with cancer may not be appropriate because children with cancer may under-report their depressive/anxiety symptoms (Challinor et al., 2000). Similarly, a review examining the prevalence of PTSS (Bruce, 2006) found that moderate to severe symptoms are present in about 5–20% of survivors. The different percentages of prevalence of PTSS among the several studies could be affected by differences in sample sizes and age, the assessment instruments used, cultural background, and the state of the disease (Ozono et al., 2007), and also by the probability that long-term physiological and psychological sequelae caused by the treatments might not manifest until pediatric survivors become adults (Prasad et al., 2010).

Other studies showed that, although most of adolescents and young adults (AYA) childhood cancer survivors were well-adjusted, in early adulthood they were more likely to have full or partial post-traumatic stress disorder (PTSD) and experience related significant functional impairment when compared with healthy peers (Schwartz and Drotar, 2006) or with siblings (Stuber et al., 2011; Varela et al., 2013).

### Predictive Factors of Post-traumatic Stress Symptoms

Factors that have been found to constitute potential risk and/or resilience factors in the development and maintenance of cancer-related PTSS can be categorized as stable (fixed and not modifiable) predictors (e.g., demographics, cancer type, physical late effects, prior life events); modifiable/dynamic (fluid and changeable) predictors (e.g., appraisal of cancer, perception of treatment, family functioning, coping styles, social support); and relational predictors (parent–child factors; Bruce, 2006). Bruce’s model of potential risk factors and/or resilience factors was adopted as our theoretical framework to meet our study aims, because it allowed us to screen the AYA pediatric survivors more at risk of developing PTSS, and also to identify the possible resource factors related to PTG.

The literature identified the following socio-demographic stable risk predictors associated with PTSS: older age at diagnosis (Hobbie et al., 2000); female gender, where females were 19% more likely than males to demonstrate significant symptoms of PTSD; single or unmarried (Stuber et al., 2011); non-white race and lower education (Smith et al., 2008); low annual income, and unemployment (Stuber et al., 2010). Some disease-related factors can be predictive of persistent symptoms: the quantity of functional/physical late effects (Brown et al., 2003; Landolt et al., 2003), active disease, more recent diagnosis, more comorbidity (Smith et al., 2008), time off treatment [although the literature on this last aspect is controversial (Langeveld et al., 2004 vs. Best et al., 2001)]. Taylor et al. (2012) found PTSS not to be related to diagnosis or treatment, but only to female gender and more reported physical late effects. A history of stressful life events in the child’s life also emerged as a salient correlate of PTSS (Currier et al., 2009); however, another study did not confirm this association (Barakat et al., 2000).

Modifiable risk factors that were independently associated with PTSD, using multiple linear regression, included less perceived social support (Langeveld et al., 2002), negative appraisals of life threat and treatment intensity, more employment and insurance issues. Additionally, several modifiable/dynamic predictors associated with PTSS included: negative subjective appraisal of life threat, less adaptive health beliefs (Taïeb et al., 2003; Schwartz et al., 2012), poor family functioning (Alderfer et al., 2009), and low family support (Brown et al., 2003). Interpersonal relationships with peers and family were identified as key predictors of a good health- related quality of life, and of psycho-social well-being, for childhood cancer survivors (Teall et al., 2013; Orbuch et al., 2015). A lower amount of satisfaction with social support was reported in girls compared with boys (Zeltzer et al., 2009; Zebrack et al., 2012) and fewer romantic relationships for those who had not undergone hematopoietic stem cell transplantation as part of their illness treatment protocol (Berbis et al., 2013).

### Post-traumatic Growth

Recent studies have suggested that this traumatic experience can also lead to positive outcomes, including PTG (Michel et al., 2009; Arpawong et al., 2013). The concept of PTG can be defined as a positive psychological change experienced as a result of a struggle with highly challenging life circumstances; in this case, a traumatic cancer experience. Most research findings related to positive consequences of childhood cancer indicate that survivors do not differ from comparison groups (Mattsson et al., 2008). Barakat et al. (2006) described adolescent survivors’ reports of positive changes in themselves, their relationships, and their life goals following successful treatment for childhood cancer. Kamibeppu et al. (2010) reported the status of PTG among young adult survivors and their siblings, showing how they had significantly more PTSS and remarkably greater PTG when compared to general controls, especially females. A recent review of qualitative and quantitative instruments assessing PTG (Duran, 2013), underlined some specific inner growth dimensions: meaning-making, life, self, family, and others. The process of meaning-making involves efforts to understand the appraised meaning of an event and to restore or incorporate that understanding into one’s global meaning system when it is disrupted or violated (Park and Folkman, 1997). A deeper sense of appreciation of life was related to existential growth, new values and life priorities. Greater self-knowledge meant a sort of overcame their weaknesses and enhancement self-improvement. A greater sense of closeness and family togetherness was related to stronger feelings of attachment and deep appreciation that children cancer survivors said they felt for their families; the children were extremely grateful for the support given to them during their cancer treatment.

A recent study (Yi and Kim, 2014) found important socio-demographic and medical correlates of PTG, such as older age and shorter time since diagnosis, as well as overall linear effects of PTSD on the PTG index. From a theoretical perspective, another study on a large sample (Klosky et al., 2014) showed a weak positive statistically significant association between PTSS and PTG, showing how these constructs might be considered as largely independent.

Arpawong et al. (2013) showed also that PTG was significantly lower among survivors of bone tumors in comparison to survivors of other cancers, and it was not significantly related to age, gender, optimism, cancer treatment, duration of treatment, or treatment intensity.

### Study Aims and Hypotheses

In this retrospective study, we aimed to identify the prevalence and severity of PTSS in a group of Italian childhood cancer survivors, considering that the literature shows considerable variability of moderate to severe PTSS percentages (5–20%; Bruce, 2006). We expected to replicate these findings in the Italian population, with about 20% of AYA childhood cancer survivors showing levels of clinical or sub-clinical symptomatology. We wanted to explore the prevalence of the PTSS criteria (intrusion, hyper-arousal, re-experience).

We aimed to identify the relative impact of three types of independent variables on the psychological well-being of childhood cancer survivors. The independent fixed variables we considered were: (1) Medical variables: type of cancer, age at diagnosis, years from the stop therapy, type of diagnosis, transplantation; (2) AYA fixed variables: age, gender, family socio-economic status, schooling years, type of job, hours per week working. The independent modifiable variable used was perceived social support with its scales: global social support, support from special other, support from friends, and support from family. The dependent variables considered were the AYA PTSS and PTG indexes.

According to the existing literature cited above, we expected that the following would self-score more PTSS: females, survivors with lower education, less time off treatment, more functional/physical late effects, low income, less perceived support, and older age at diagnosis.

We aimed also to gain from the PTG scores quantitative information, not only on the possible psycho-pathological aspects, but also on the resilience factors related to PTG. For this purpose, we wanted to estimate the relationship between PTSS and PTG in childhood cancer survivors who had stopped therapy for at least 5 years, and to relate these constructs to other socio-demographic and illness variables. We expected PTG to be related to PTSS (Yi and Kim, 2014), with a weak positive statistical significance (Klosky et al., 2014). We expected that older age and shorter time since stopping therapy would be associated with PTG (Yi and Kim, 2014); and we wanted to explore other possible predictive factors, for example, perceived social support.

## Materials and Methods

### Participants

Participants comprised 223 AYA cancer survivors recruited during follow-up visits in the Oncohematology Clinic of the Department of Child and Women’s Health, University of Padua. Self-report questionnaires were administered to gain data on PTSS incidence, PTG, perceived social support, and medical and socio-demographic factors. Ex-patients’ mean age at the assessment was 19.33 years (*SD* = 3.01, range: 15–25), 123 males and 100 females, with a mean of years off-therapy of 9.64 (*SD* = 4.17, range: 5–24). Most (52.5%) had survived an hematological disorder, and 47.5% a solid tumor when they were aged an average of 8.02 years (*SD* = 4.40, range: 0.01–17.03). **Table 1** illustrates the socio-demographic and medical data of survivors.

**Table 1:** Socio-demographic and clinical characteristics of the sample.

		*N*	%
Gender	Male	123	55.2
	Female	100	44.8
Age	15–17 years	81	36.3
	18–21 years	88	39.5
	22–25 years	54	24.2
Education	8 years of schooling	95	42.6
	13 years of schooling	103	46.2
	Degree	17	7.6
	*Missing*	8	*3.6*
Relationship status	Engaged	63	28.3
	Single	99	44.4
	*Missing*	*61*	*27.4*
Diagnosis type	Hematological disorders	115	51.57
	Acute myeloid leukemia	11	
	Acute lymphoblastic leukemia	74	
	Chronic myeloid leukemia	2	
	Non-Hodgkin lymphoma	28	
	Solid tumors	108	48.43
	Hepatoblastoma	1	
	Hodgkin lymphoma	37	
	Langerhans cell histiocytosis	6	
	Neuroblastoma	8	
	Bone tumor	5	
	Ovarian tumor	1	
	Rhabdomyosarcoma	12	
	Retinoblastoma	1	
	Soft tissue sarcoma	9	
	Wilms tumor	20	
	Others	8	
Transplantation	Yes	32	14.3
	No	191	85.7
Job (*N* = 85)	Looking for a job	20	24.1
	Part-time	14	16.9
	Full-time	49	59.0
		**Mean (range)**	***SD***
Age at diagnosis, years		8.02 (0.01–17.03)	4.40
Time from stop therapy, years		9.65 (5–24)	4.17

### Procedure

All eligible survivors attending the Pediatric Hematology-Oncologic Clinic, University of Padua, from October 2008 to September 2012, were asked to take part in this study. Eligibility criteria included treatment for cancer before the age of 18 years, at least 5 years out of therapy, and currently aged 15–25 years. We excluded survivors treated for central nervous system tumors, or with learning, or sensory problems, or with genetic syndromes that made them unable to complete questionnaires alone, and those that did not reach the inclusion criteria at the appointment time (i.e., the age range, or at least 5 years out of therapy). We also excluded also survivors with any cognitive and learning disabilities, because these cognitive limitations would have not allowed them to fill in the self-report questionnaires.

In all, 230 of 296 eligible survivors entered the study. The patients who did not participate were not contacted for this study because they had no regular or further follow-up visits, or they had changed address. Completed questionnaires were received from 223 individuals (response rate: 96.95%).

Ethics approval was obtained from the local ethics committees. The day before the follow-up appointment at the day hospital of the Clinic, the clinical psychologist telephoned each survivor to explain the study and to obtain consent to participation for the next day. If the survivor was less than 18 years-old, the parent was contacted before talking to the child. On arrival at the clinic, an information pack that included information about the study, a consent form and some self-reported questionnaires were given to participants. Completed consent forms were obtained from the ex-patients or from the parents of minors. The other questionnaires were returned to us in stamped addressed envelopes, or electronically via a protected online site.

The questionnaires comprised the PTSD Symptom check-list, the personal growth questionnaire that was developed from the childhood cancer survivor study (CCSS; available on the CCSS website) and the multidimensional scale of perceived social support (MSPSS; Zimet et al., 1990). Medical and socio-demographic data were also collected.

#### The Post-traumatic Stress Disorder Symptom Check-List

The PTSD symptom check-list is a 17 items-check-list assessing the presence of symptoms of PTSD that may arise after a very stressful situation, such as the experience of cancer. It is an adapted version (Manne et al., 1998) of the instrument used in the CCSS (Zebrack, 2008; Stuber et al., 2010), and contains a list of possible problems related to the traumatic cancer experience.

Adolescent and young adult pediatric cancer survivors filled in the questionnaire about the presence/absence (no/yes, score 0/1) of these problems in their lives occurring within a month after the communication of the diagnosis. These 17 items were divided into categories or symptoms related to intrusion (five items), avoidance (seven items) and arousal (five items; DSM IV guided this categorization). Cut-off scores for symptom severity were ≤5 = not diagnostic of the disorder; 6–8 = moderate presence of the disorder; >9 = marked severity (an adaptation of PTSD-RI, Frederick, 1985). Using the full continuum of PTSS from mild to severe, as assessed by self-report, may be more informative than using PTSD as a dichotomous variable.

Manne et al. (1998) demonstrated good reliability and validity indices for the instrument. The reliability of the Italian version PTSD symptom inventory was also assessed and demonstrated good internal consistency (Cronbach α = 0.72; Tremolada et al., 2013).

#### The Personal Growth Inventory

The personal growth inventory is a 21 items questionnaire assessing the influence of illness (score: 0–5) on life perceptions and priorities of AYA surviving cancer. This survey assessed the extent to which respondents believed they had been influenced by their cancer experience. Participants were asked to respond to each item using a six-point scale, ranging from 0 (“I am NOT influenced by my experience”) to 5 (“I am influenced to a VERY GREAT degree by my experience”). Personal growth inventory total scores range from 0 to 105, with higher scores suggesting greater PTG.

A Varimax rotated confirmatory factor analysis was run to identify the possible factors of personal growth. The results on this sample of childhood cancer survivors showed that three factors could be extracted according to Kaiser’s rule, explaining overall a good proportion of the total variance (65.35%). The factors were: perceptions of life and myself (*N* = 8 items; α = 0.91; 27.12 of variance); Relationships and openness toward the world (*N* = 7 items; α = 0.89; 21.77 of variance); Spirituality/religion and change (*N* = 5 items; α = 0.75; 10.46 of variance); and Impulsivity (*N* = 4 items, α = 0.84; 16.45 of variance). Internal consistency of the sub-scales was good. Correlations among the factors showed that the scales were moderately independent from each other (range: 0.77–0.80).

#### The Multidimensional Scale of Perceived Social Support

The MSPSS (Zimet et al., 1990) is a 12 Likert-style items questionnaire measuring the instrumental and emotional social support provided by family, friends, and significant others. The scale is based on a range of 1 (not at all) to 5 (very much). A total score of 12 denotes low social support; 84, a high level. Participants were asked to respond to the questionnaire in terms of the social support they received. The Cronbach alpha reliability, as determined by its authors, was found to be high (0.85–0.95), with a test-retest reliability of *r* = 0.85. The questionnaire was also found to have construct validity, as determined by factor analysis in two separate studies (Zimet et al., 1990; Clara et al., 2003) and content validity (Bruwer et al., 2008). Internal consistency reliability for the current sample was 0.93 for the total score and, regarding the scales, 0.94 (significant other), 0.89 (friends) and 0.91 (family).

### Statistical Methods

Descriptive statistics were calculated to understand the distribution of the PTSS severity and the PTG indexes in our sample. We ran preliminary Pearson bivariate correlations to find significant associations between the examined variables in order to identify the relevant variables to insert in the hierarchical linear regression analysis, and to test the possible associations between PTSS and PTG. Then, a series of hierarchical linear regression analyses were run to identify predictors of PTSS and PTG outcomes in childhood cancer survivors. In the first regression step, we inserted the following independent fixed variables: gender, actual age, age at diagnosis, years off-therapy, relationship status (single = 1 vs. engaged = 2), and years of education. In the second step, the following variables related to perceived social support were inserted: support from significant other, support from family, support from friends, and global support score. We excluded the following variables that did not emerge in the preliminary correlations as significant: type of diagnosis (hematologic vs. solid tumor), transplantation, and the perceived socio-economic condition (low, medium, high). The dependent variables inserted one at a time were the three PTSS criterion scales, the PTSS global index, and all the scores dealing with PTG. Statistical significance was set at the nominal *p* = 0.05 level, with adjustments for multiple comparisons. The effect size in the regression analyses was assessed according to Cohen’s rules (Cohen, 1988).

## Results

### Frequencies of Post-traumatic Stress Disorder Symptomatology, Post-traumatic Growth Scores, Perceived Social Support and their Relationships

**FIGURE 1:**
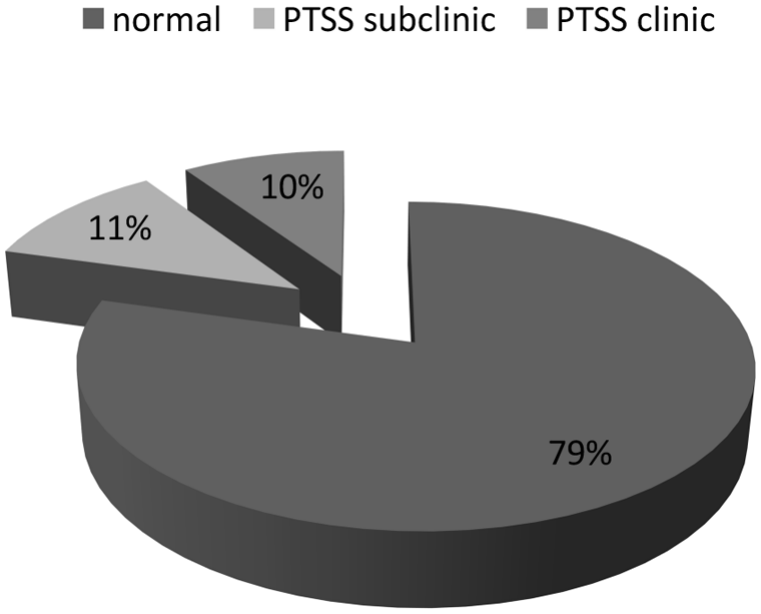
**Post-traumatic stress symptom (PTSS) presence in childhood cancer survivors**.

The results indicated a moderate presence of clinical (≥9 symptoms: 9.4%) and sub-clinical (6–8 symptoms: 11.2%) PTSS. The avoidance criterion (mean = 1.34; *DS* = 1.64; range: 0–7) was most often present, followed by hyper-arousal symptoms (mean = 1.31; *SD* = 1.35; range: 0–6). The re-experience criterion (mean = 0.95; *SD* = 1.34; range: 0–7) was the least often reported (**Figure 1**).

The mean of the PTG global index was 3.15 (*SD* = 1.25) and the means of the sub-scales were as follows: 3.44 (*SD* = 1.39) for Life and myself perceptions, 2.99 (*SD* = 1.32) for Relations and openness toward the world and 2.64 (*SD* = 1.25) for Spirituality/religion and Change. Perceived social support was attested at a medium level regarding global support (mean = 36.64; *SD* = 10.67; range: 0–60). Support from family (mean = 13.03; *DS* = 16.89; range: 0–20) was the higher score, followed by Support from significant other (mean = 12.27; *DS* = 4.74; range: 0–20) and Support from friends (mean = 11.39; *DS* = 4.09; range: 0–20).

Pearson correlations identified significant associations between PTG and PTSS (*r* = 0.15; *p* = 0.02; *N* = 216) and between PTG and the Re-experience of PTSS symptoms (*r* = 0.24; *p* = 0.0001; *N* = 216), while no significant association was found between PTG respectively with the Avoidance (*r* = 0.10; *p* = 0.15) and Hyper-arousal symptoms criteria (*r* = 0.10; *p* = 0.15). We then calculated statistical significance with adjustments for multiple comparisons, taking as significant a *p*-value of ≤0.01. Only the association between PTG and the Re-experience of PTSS symptoms could be accepted as significant, with a medium effect size of *r*, according to Cohen’s rules.

### Models of Stable and Modifiable Predictive Factors Related to Post-traumatic Stress Symptoms

To verify which socio-demographic, disease factors and type of perceived social support predict PTSS in childhood cancer survivors, we ran a hierarchical regression analysis. The model that resulted the best fit (*R*^2^ = 0.08; *F* = 1.46; *p* = 0.05) showed Gender (β = 0.16; *p* = 0.05) and the Support global score (β = -0.43; *p* = 0.05) impacting upon the PTSS global index.

Other models were run for each PTSS criterion symptoms scale, with the same independent variables inserted in the two steps regression analysis. The first model with the fixed independent variables (socio-demographic and illness variables) was in the best fit for predicting Re-experience of PTSS scale (*R*^2^ = 0.06; *F* = 2.55; *p* = 0.02), with only Gender (β = 0.16; *p* = 0.04) impacting on the Re-experience criterion.

The second model with fixed and modifiable variables was in the best fit for predicting the Avoidance PTSS scale (*R*^2^ = 0.11; *F* = 2.06; *p* = 0.03), with the Relationship status (β = 0.18; *p* = 0.04) and Support global score (β = -0.48; *p* = 0.02) as the significant predictive factors.

**FIGURE 2:**
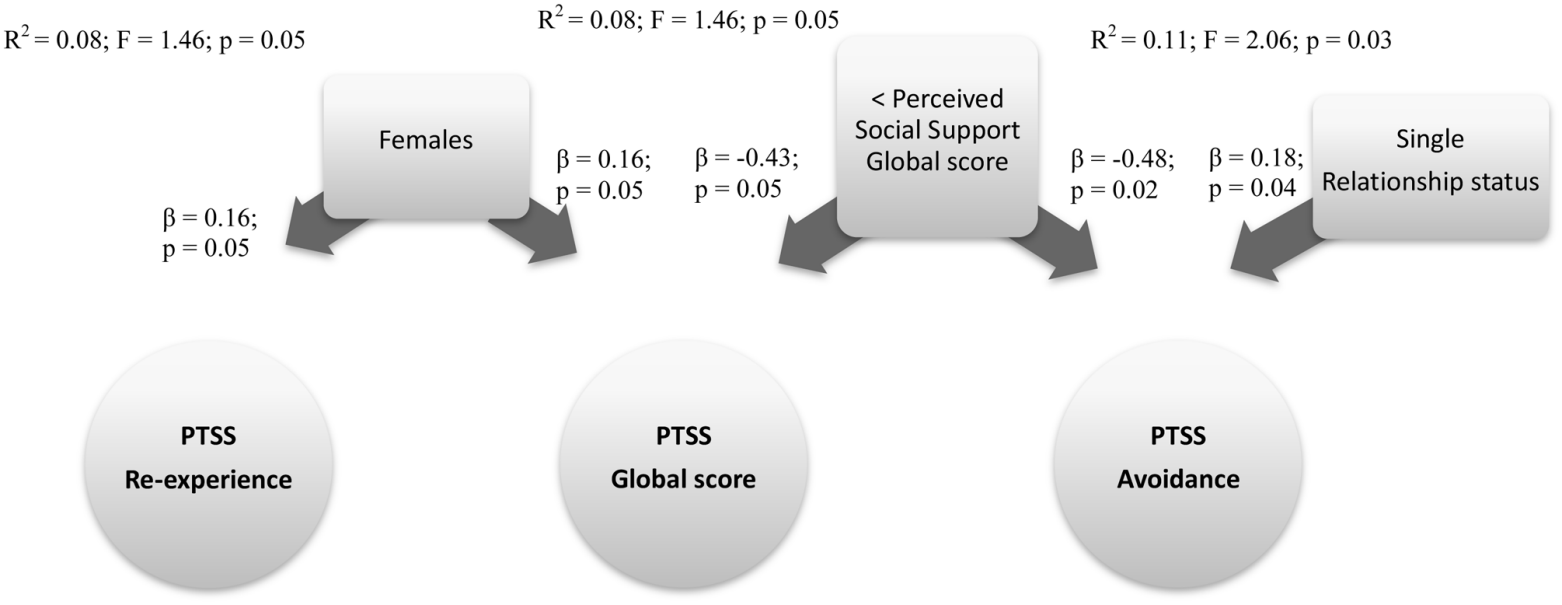
**Predictive factors of PTSS global score, PTSS re-experience, and avoidance criterions**. Rectangle: stable Independent variables; Square: modifiable Independent variables; Circle: Dependent variables.

We note that all the *R*^2^ of these models explained only a small part of variance, and point out that other factors not assessed here could impact on these PTSS indexes. However, these results can give us a clinical indication. **Figure 2** shows a summary of these three models.

### Models of Stable and Modifiable Predictive Factors Related to Post-traumatic Growth

Which socio-demographic and disease factors, and what type of perceived social support are responsible for PTG in childhood cancer survivors?

**FIGURE 3:**
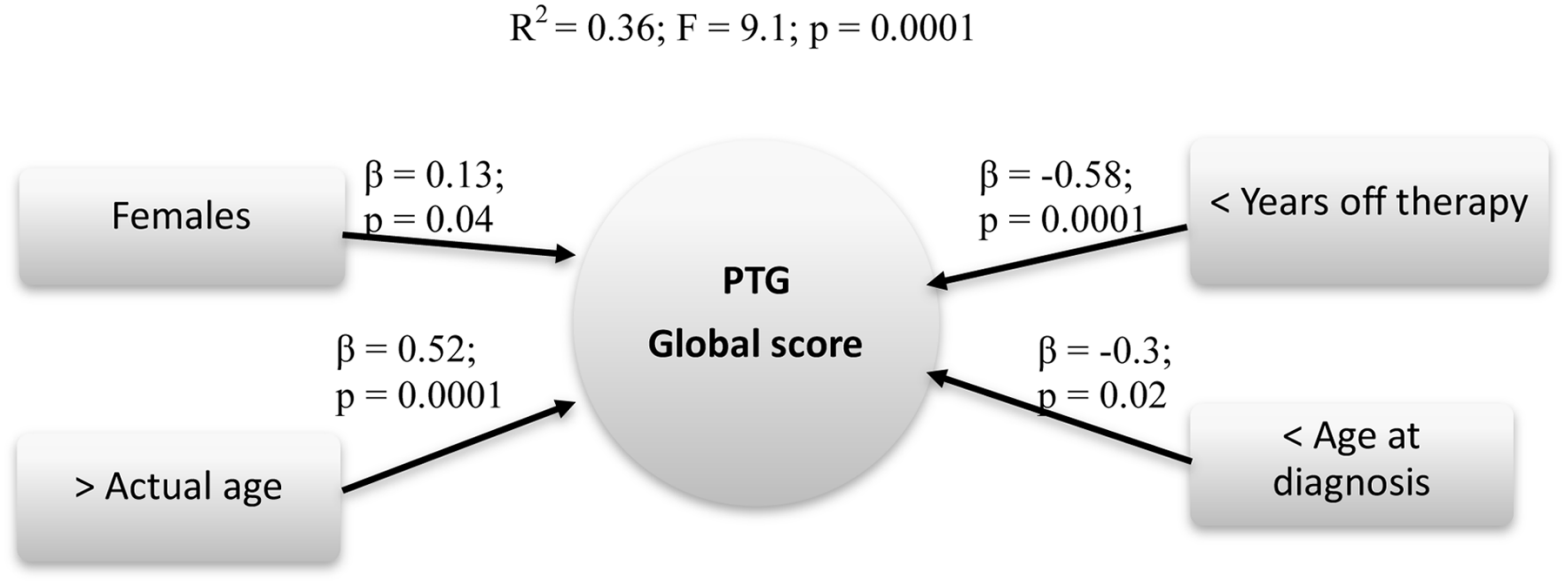
**Predictive factors of PTG global score**. Rectangle: stable Independent variables; Circle: Dependent variables

To answer this question, we conducted a hierarchical regression analysis to assess the possible predictors of PTG total score. The second model (fixed and modifiable independent variables inserted) resulted the best fit (*R*^2^ = 0.36; *F* = 9.1; *p* = 0.0001), with Gender (β = 0.13; *p* = 0.04), Actual age (β = 0.52; *p* = 0.0001), Age at diagnosis (β = -0.3; *p* = 0.02) and Years off-therapy (β = -0.58; *p* = 0.0001) impacting upon the PTG global index (**Figure 3**).

The same models were run for each PTG scale. The best fit model for predicting Life and Myself perceptions (*R*^2^ = 0.32; *F* = 7.6; *p* = 0.0001) identified the following factors as being the best predictors: Gender (β = 0.14; *p* = 0.04), Actual age (β = 0.46; *p* = 0.0001), Age at diagnosis (β = -0.26; *p* = 0.05), Years off-therapy (β = -0.49; *p* = 0.0001), and Support from family (β = 0.23; *p* = 0.03).

Relations and openness toward the world (*R*^2^ = 0.31; *F* = 7.25; *p* = 0.0001) was predicted by Actual age (β = 0.49; *p* = 0.0001), Age at diagnosis (β = -0.33; *p* = 0.04), and Years off-therapy (β = -0.56; *p* = 0.0001).

Finally, Spirituality/religion and Change (*R*^2^ = 0.2; *F* = 6.95; *p* = 0.0001) increased by Gender (β = 0.16; *p* = 0.03), Actual age (β = 0.46; *p* = 0.0001), Age at diagnosis (β = -0.33; *p* = 0.02), Years off-therapy (β = -0.53; *p* = 0.0001), and Schooling years (β = 0.17; *p* = 0.05).

**FIGURE 4:**
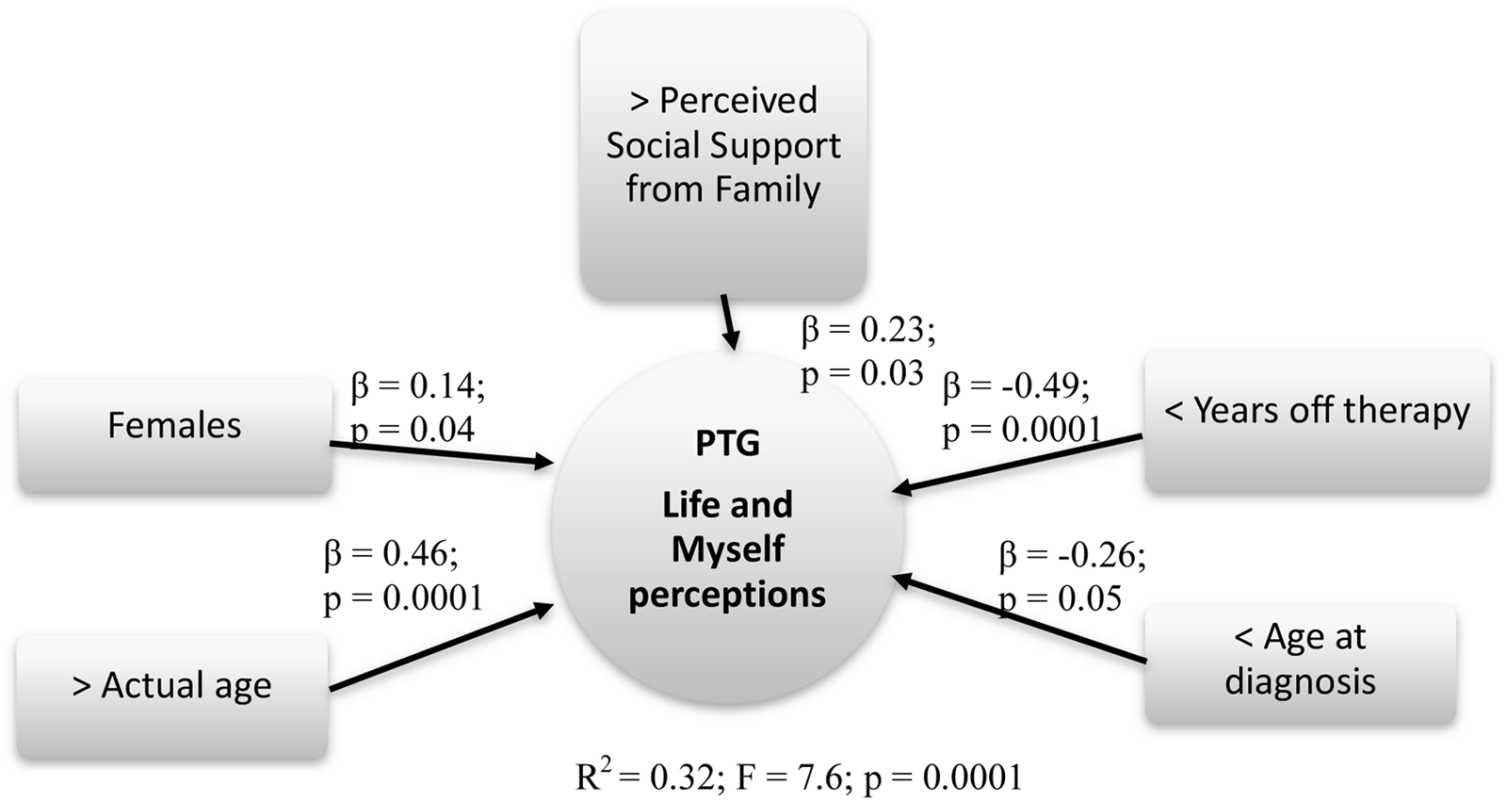
**Predictive factors of PTG life and myself perceptions**. Rectangle: stable Independent variables; Square: modifiable Independent variables; Circle: Dependent variables.

**FIGURE 5:**
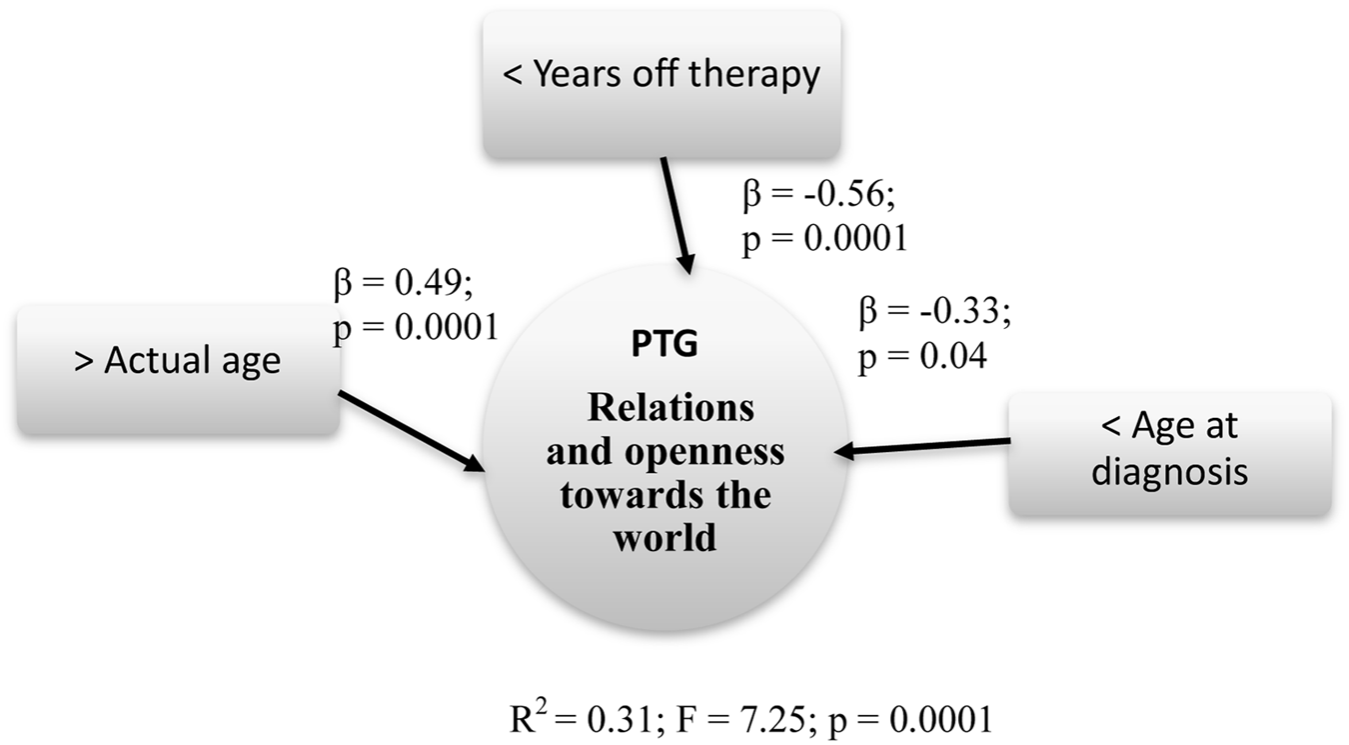
**Predictive factors of PTG relations and openness toward the world**. Rectangle: stable Independent variables; Square: modifiable Independent variables; Circle: Dependent variables.

**FIGURE 6:**
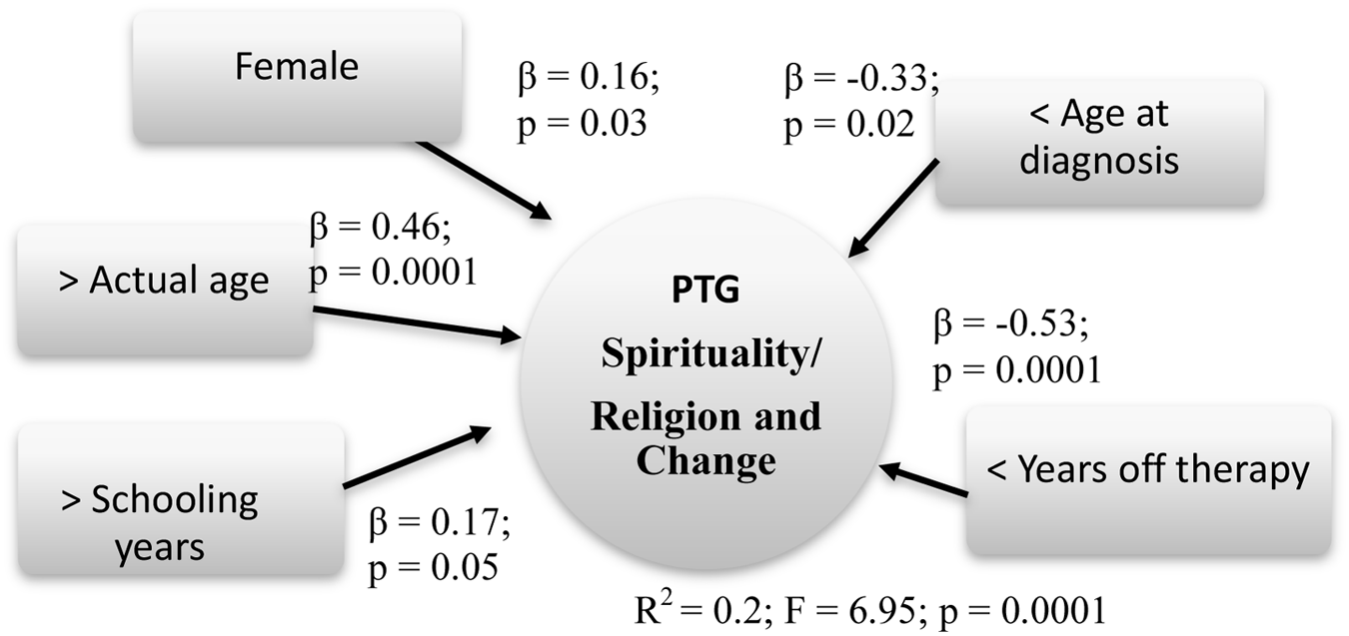
**Predictive factors of PTG spirituality/religion and change**. Rectangle: stable Independent variables; Square: modifiable Independent variables; Circle: Dependent variables

Summary models of predictive factors related to PTG are presented respectively in **Figures 4–6**.

We note that all the *R*^2^ of these models explained a medium part of variance, and point out that the factors we have identified largely impacted on PTG scores.

## Discussion

An important long-term effect on AYA pediatric cancer survivors’ well-being could be the symptomatology associated with PTSD (Bruce, 2006; Ozono et al., 2007; Smith et al., 2008). PTSSs, such as intrusive thoughts about past illness or particularly stressful moments during treatment, physiological arousal at reminders of cancer, and avoidance of thoughts concerning cancer and its treatment, were closely linked to follow-up and continued to be common among AYA cancer survivors healed for many years. Other studies (Bruce, 2006; Smith et al., 2008) revealed a prevalence of PTSS in about 5–20% of survivors. In our Italian group of AYA pediatric cancer survivors, the prevalence of PTSS was about 21%. Specifically, 10% of our ex-patients belonged to a clinical category, while 11% could be classified as sub-clinical. The avoidance criterion was most often met, followed by hyper-arousal symptoms and the re-experience criterion.

Following the model of Bruce (2006) as the theoretical framework of this study, the fixed factor of female gender was confirmed as a strong predictor of developing severe PTSS (Hobbie et al., 2000; Langeveld et al., 2004), especially regarding re-experience symptoms. Following this model, other fixed variables, such as off-therapy years (Langeveld et al., 2004), low income situation (Stuber et al., 2010), less education (Smith et al., 2008) and older age at diagnosis (Hobbie et al., 2000), did not emerge as significant predictors. An important modifiable predictor protecting from PTSS severity was identified as the global perceived social support reported by the healed patients, as some other studies have found (Langeveld et al., 2002; Teall et al., 2013; Orbuch et al., 2015). In this study, the single relationship status was a risk predictor of Avoidance PTSS, confirming how having fewer relationships with others impacted negatively on psychological well-being. These AYA cancer survivors declared a medium level of perceived social support, with a preference for family support that had a key role in helping them during their illness experience and in their routine daily lives.

Post-traumatic growth is another important issue, recently studied in the literature, that can be defined as a positive psychological change resulting from a struggle with highly challenging life circumstances, that is, a traumatic cancer experience (Duran, 2013). A recent study (Yi and Kim, 2014) identified a linear effect of PTSD on the PTG index. PTSD and PTG were associated with each other; these are two sides of the same coin, the cancer traumatic experience, but this linearly significant association is statistically valid and recognizable only for the Re-experience of PTSS criterion.

The factor analysis of the PTG questionnaire revealed three important dimensions, as follows: a new point of view on self and on life in general; different perspectives on their relationships with other individuals and toward the world; and a new pathway in their spirituality. These dimensions also emerged in other studies. For example, Barakat et al. (2006) described adolescent survivors’ reports of positive changes respectively in themselves (our first dimension), in their relationships (our second), and their life goals. In the recent study of Duran (2013), the five specific inner growth dimensions identified (meaning-making, appreciation of life, self-awareness, closeness and family togetherness, and a desire to pay back society), can also be found throughout our three factor model. A deeper sense of appreciation of life, new values and life priorities, and a greater self-knowledge for overcoming their weaknesses and enhancing self-improvement could be identified in the first factor. Another factor dealt with a greater sense of closeness and family togetherness and a desire to pay back society; these we could find in our second factor.

### But Which Factors Influenced the PTG?

A recent study (Yi and Kim, 2014) identified older age and shorter time since diagnosis as important correlates of PTG. Our data confirmed these results and also identified other predictive factors, such as female gender and younger age at diagnosis. The same factors (older actual age, shorter time since off-therapy, female gender, younger age at diagnosis) were predictors for the dimension Life and myself perceptions; however, another innovative and important modifiable factor was found: the perceived support from family reported by healed patients. Trauma could be turned into a personal growth experience in their own lives and in their self-perceptions if they had family support that helped them throughout the cancer treatments and in their daily routine re-entry processes.

Regarding the dimension Relations and openness toward the world, the factors that impacted more were as follows: minor age at diagnosis, less off-therapy years, and older age at the study. The Spirituality/religion and Change dimension was influenced by the same factors of the PTG global score, adding also the higher schooling years.

The factors that influenced PTSS were not the same as those affecting the PTG scores and sub-scales; these constructs were associated but were not identical.

As survival rates improve, follow-up in pediatric clinics becomes less feasible, and alternative models of care have been proposed to determine links between treatment and late effects, and to screen and inform individuals so as to provide timely intervention if problems arise (Absolom et al., 2006; Michel et al., 2011). Based on these empirical results, specific psychological interventions could be devised for the childhood cancer survivors more at risk of developing PTSS; that is, females who report less global social support from a special other, from friends and from family. We know that adolescents experience many stressor situations, ranging from pubertal changes to family and peer relationship conflicts. Facing cancer, the stress can become unbearable and could result in psychological symptoms that could threaten their psychological health. A female adult survivor aged 20 years-old explained this concept in a clinical interview:

“*Well, I think I’ve blocked a lot of my adolescence out, in the sense that, because it happened in a period of transition. I came to the secondary school, in the second year, I knew no-one, that is, in short, it was a bit tragic, in fact I have a very bad memory of that period and up to the third year of school there was no way to unblock…they were not good years in terms of relational [...] that is, I always felt a bit looked at by the other people*.”

Perhaps for this reason, older age at diagnosis was associated with minor PTG. Traumatic experience at this age was not strictly associated with the actual severity of PTSS, but rather to the possibility of gaining personal growth from it. PTG could help the survivors to cope with their traumatic experience within only a few years from stopping treatment. Clinical intervention could focus on the adolescents and young adults, talking with them about their social and romantic relationships, what the illness meant for them, and what had been added or erased from their lives. A narrative technique could be useful to them to make sense of their illness experience, along with the use of therapeutic group intervention, so that they feel less isolated by sharing their experiences with other cancer survivors. Family could also help the healed patients by their support; psychologists could give them some clinical advice to support their children and adolescents in their new daily life, without being too protective.

Adolescents and young adults with less years off-therapy probably develop fresh psychological coping strategies to adapt to the short and long-term effects of this traumatic experience. Furthermore, and especially if more scholastically advanced, they could ascribe a different meaning to their illness, with an increase in their spirituality related to the concept of the post-traumatic personal growth. For example, a male survivor aged 24 years, narrating his experience, told us that

“*These are the worlds that you see only when you have passed through them, because after getting out of this experience, you can understand that other problems, other people have problems, and then you give them a hand, they’ll thank you and this is important.”*

There are some limitations of this study. Only a north-east Italian clinic population was involved; the predictive models on PTSS explained only a small part of variance, stressing that other factors not assessed here could impact on the PTSS indexes; and the self-reported questionnaires were subjected to social desirability and they could not be completely objective measures. We decided to exclude ex-patients with learning disabilities or mental retardation at the diagnosis time because their cognitive limitations would have not allowed them to fill in the self-report questionnaires. However, learning disabilities could constitute a post-traumatic consequence of the illness (even if, in our group of patients, no new case of learning disabilities was identified after the cancer treatment), so future research to clarify this point is necessary.

Our future direction for research will involve using the in-depth interviews that we carried out in this study to assess the possible associations of the questionnaires with the narratives. Another important future direction could be to gather information about the patient and/or the family psychological support received during their illness experience, and also in the off-therapy phase. A recent study underlined how the parental, especially the maternal, PTSS might be a risk factor for the child’s PTSS (Morris et al., 2012). Consequently, recommendations for future research would include obtaining information about the parents’ psychological functioning that could influence their child’s well-being, both during and after therapy.

The strengths of this study include the innovative use of these survey instruments for this clinical population in our Italian context and their good psychometric properties, the high number of survivors, the selection criteria of the sample and the innovative assessment of PTG and its specific scales.

## Author Contributions

MT conceptualized and designed the study, carried out the analyses, reviewed and revised the manuscript, drafted the initial manuscript, and approved the final manuscript as submitted. SB coordinated and supervised data collection, critically reviewed the manuscript, and approved the final manuscript as submitted. MP and GB provided local in the Clinic, supported the data collection phase and the study, approved the final manuscript as submitted. All authors approved the final manuscript as submitted and agree to be accountable for all aspects of the work.

## Conflict of Interest Statement

The authors declare that the research was conducted in the absence of any commercial or financial relationships that could be construed as a potential conflict of interest. The reviewer FL and handling Editor declared their shared affiliation, and the handling Editor states that the process nevertheless met the standards of a fair and objective review.

## References

[B1] Absolom K. Greenfield D. Ross R. Horne B. Davies H. Glaser A. (2006). Predictors of clinic satisfaction among adult survivors of childhood cancer. *Eur. J. Cancer* 42 1421–1427. 10.1016/j.ejca.2006.01.053 16759851

[B2] Alderfer M. A. Navsaria N. Kazak A. E. (2009). Family functioning and posttraumatic stress disorder in adolescent survivors of childhood cancer. *J. Fam. Psychol.* 23 717–725. 10.1037/a0015996 19803607 PMC2777540

[B3] Arpawong T. E. Oland A. Milam J. E. Ruccione K. Meeske K. E. (2013). Post-traumatic growth among an ethnically diverse sample of adolescent and young adult cancer survivors. *Psychooncology* 22 2235–2244. 10.1002/pon.3286 23554227 PMC8723731

[B4] Barakat L. Kazak A. E. Gallagher M. A. Meeske K. Stuber M. L. (2000). Posttraumatic stress symptoms and stressful life events predict the long-term adjustment of survivors of childhood cancer and their mothers. *J. Clin. Psychol. Med. Settings* 7 189–196. 10.1023/A:1009516928956

[B5] Barakat L. P. Alderfer M. A. Kazak A. E. (2006). Posttraumatic growth in adolescent survivors of cancer and their mothers and fathers. *J. Pediatr. Psychol.* 31 413–419. 10.1093/jpepsy/jsj058 16093518

[B6] Berbis J. Michel G. Chastagner P. Sirvent N. Demeocq F. Plantaz D. (2013). A French cohort of childhood leukemia survivors: impact of hematopoietic stem cell transplantation on health status and quality of life. *Biol. Blood Marrow Transplant.* 19 1065–1072. 10.1016/j.bbmt.2013.04.015 23618717

[B7] Best M. Streisand R. Catania L. Kazak A. E. (2001). Parental distress during pediatric leukemia and posttraumatic stress symptoms (PTSS) after treatment ends. *J. Pediatr. Psychol.* 26 299–307. 10.1093/jpepsy/26.5.299 11390572

[B8] Brown R. T. Madan-Swain A. Lambert R. (2003). Posttraumatic stress symptoms in adolescent survivors of childhood cancer and their mothers. *J. Trauma Stress* 16 309–318. 10.1023/A:1024465415620 12895012

[B9] Bruce M. (2006). A systematic and conceptual review of posttraumatic stress in childhood cancer survivors and their parents. *Clin. Psychol. Rev.* 26 233–256. 10.1016/j.cpr.2005.10.002 16412542

[B10] Bruwer B. Emsley R. Kidd M. Lochner C. Seedat S. (2008). Psychometric properties of the multidimensional scale of perceived social support in youth. *Compr. Psychiatry* 49 195–201. 10.1016/j.comppsych.2007.09.002 18243894

[B11] Challinor J. Miaskowski C. Moore I. Slaughter R. Franck L. (2000). Review of research studies that evaluated the impact of treatment for childhood cancers on neurocognition and behavioral and social competence: nursing implications. *J. Soc. Pediatr. Nurs.* 5 57–74. 10.1111/j.1744-6155.2000.tb00088.x 10879361

[B12] Clara I. P. Cox B. J. Enns M. W. Murray L. T. Torgrudc L. J. (2003). Confirmatory factor analysis of the multidimensional scale of perceived social support in clinically distressed and student samples. *J. Pers. Assess.* 81 265–270. 10.1207/S15327752JPA8103_09 14638451

[B13] Cohen J. (1988). *Statistical Power Analysis for the Behavioural Sciences.* Hillsdale, NJ: Erlbaum.

[B14] Currier J. M. Jobe-Shields L. E. Phipps S. (2009). Stressful life events and posttraumatic stress symptoms in children with cancer. *J. Trauma Stress* 22 28–35. 10.1002/jts.20382 19117041 PMC2649970

[B15] Duran B. (2013). Posttraumatic growth as experienced by childhood cancer survivors and their families: a narrative synthesis of qualitative and quantitative research. *J. Pediatr. Oncol. Nurs.* 30 179–197. 10.1177/1043454213487433 23657991

[B16] Eiser C. Hill J. J. Vance Y. H. (2000). Examining the psychological consequences of surviving childhood cancer: systematic review as a research method in pediatric psychology. *J. Pediatr. Psychol.* 25 449–460. 10.1093/jpepsy/25.6.449 10980049

[B17] Frederick C. J. (1985). “Selected foci in the spectrum of posttraumatic stress disorders,” in *Perspectives on Disaster Recovery*, eds Laube J. Murphy S. A. (Norwalk, CT: Appleton-Century-Crofts), 110–130.

[B18] Hobbie W. L. Stuber M. Meeske K. Wissler K. Rourke M. T. Ruccione K. (2000). Symptoms of posttraumatic stress in young adult survivors of childhood cancer. *J. Clin. Oncol.* 18 4060–4066. 11118467 10.1200/JCO.2000.18.24.4060

[B19] Homer M. J. Ries L. A. G. Krapcho M. Neyman N. Aminou R. Howlader N. (2009). *SEER Cancer Statistics Review, 1975–2006, National Cancer Institute. [Based on November 2008 SEER Data Submission, Posted to the SEER Web Site]*. Available at: http://seer.cancer.gov/csr/1975_2006/

[B20] Kamibeppu K. Sato I. Honda M. Ozono S. Sakamoto N. Iwai T. (2010). Mental health among young adult survivors of childhood cancer and their siblings including posttraumatic growth. *J. Cancer Surviv.* 4 303–312. 10.1007/s11764-101-1024-z 20396974

[B21] Klosky J. L. Krull K. R. Kawashima T. Leisenring W. Randolph M. E. Zebrack B. (2014). Relations between posttraumatic stress and posttraumatic growth in long-term survivors of childhood cancer: a report from the Childhood Cancer Survivor Study. *Health Psychol.* 33 878–882. 10.1037/hea0000076 24799000 PMC4158696

[B22] Landolt M. A. Vollrath M. Ribi K. Gnehm H. E. Sennhauser F. H. (2003). Incidence and associations of parental and child posttraumatic stress symptoms in pediatric patients. *J. Child Psychol. Psychiatry* 44 1199–1207. 10.1111/1469-7610.00201 14626460

[B23] Langeveld N. E. Grootenhuis M. A. Voûte P. A. de Haan R. J. (2004). Posttraumatic stress symptoms in adult survivors of childhood cancer. *Pediatr. Blood Cancer* 42 604–610. 10.1002/pbc.20024 15127415

[B24] Langeveld N. E. Stam H. Groothenhuis M. A. Last B. E. (2002). Quality of life in young adult survivors of childhood cancer. *Support Care Cancer* 10 579–600. 10.1007/s00520-002-0388-6 12436217

[B25] Manne L. Du Hamel K. Gallelli K. Sorgen K. Redd W. H. (1998). Posttraumatic stress disorder among mothers of pediatric cancer survivors: diagnosis, comorbidity, and utility of the PTSD Checklist as a screening instrument. *J. Pediatr. Psychol.* 23 357–366. 10.1093/jpepsy/23.6.357 9824924

[B26] Mattsson E. Lindgren B. Von Essen L. (2008). Are there any positive consequences of childhood cancer? *Acta Oncol.* 47 199–206. 10.1080/02841860701765667 18210296

[B27] Michel G. Greenfield D. Absolom K. Eiser C. (2011). Satisfaction with follow-up consultations among younger adults treated for cancer: the role of quality of life and psychological variables. *Psychooncology* 20 813–822. 10.1002/pon.1783 20878873

[B28] Michel G. Taylor N. Absolom K. Eiser C. (2009). Benefit finding in survivors of childhood cancer and their parents: further empirical support for the Benefit Finding Scale for Children. *Child Care Health Dev.* 36 123–129. 10.1111/j.1365-2214.2009.01034.x 19961498

[B29] Morris A. Gabert-Quillen C. Delahanty D. (2012). The association between parent PTSD/depression symptoms and child PTSD symptoms: a meta-analysis. *J. Pediatr. Psychol.* 37 1076–1088. 10.1093/jpepsy/jss091 23019132

[B30] Orbuch T. Parry C. Chesler M. Fritz J. Repetto P. (2015). Parent–child relationships and quality of life: resilience among childhood cancer survivors. *Fam. Relat.* 54 171–183. 10.1111/j.0197-6664.2005.00014.x

[B31] Ozono S. Saeki T. Mantani T. Ogata A. Okamura H. Yamawaki S. (2007). Factors related to posttraumatic stress in adolescent survivors of childhood cancer and their parents. *Support Care Cancer* 15 309–317. 10.1007/s00520-006-0139-1 17021857

[B32] Park C. L. Folkman S. (1997). Meaning in the context of stress and coping. *Rev. Gen. Psychol.* 1 115–144. 10.1037/1089-2680.1.2.115

[B33] Patenaude A. F. Kupst M. J. (2005). Psychosocial functioning in pediatric cancer. *J. Pediatr. Psychol.* 30 9–27. 10.1093/jpepsy/jsi012 15610981

[B34] Prasad P. K. Bowles T. Friedman D. L. (2010). Is there a role for a specialized follow-up clinic for survivors of pediatric cancer? *Cancer Treat. Rev.* 36 3723–3776. 10.1016/j.ctrv.2010.02.014 20189720

[B35] Pui C. H. Cheng C. Leung W. Rai S. N. Rivera G. K. Sandlund J. T. (2003). Extended follow-up of long-term survivors of childhood acute lymphoblastic leukemia. *N. Engl. J. Med.* 349 640–649. 10.1056/NEJMoa035091 12917300

[B36] Schwartz L. Drotar D. (2006). Posttraumatic stress and related impairment in survivors of childhood cancer in early adulthood compared to healthy peers. *J. Pediatr. Psychol.* 31 356–366. 10.1093/jpepsy/jsj018 15788716

[B37] Schwartz L. A. Kazak A. E. DeRosa B. W. Hocking M. C. Hobbie W. L. Ginsberg J. P. (2012). The role of beliefs in the relationship between health problems and posttraumatic stress in adolescent and young adult cancer survivors. *J. Clin. Psychol. Med. Settings* 19 138–146. 10.1007/s10880-011-9264-1 21964825 PMC3420807

[B38] Sloper P. (2000). Predictors of distress in parents of children with cancer: a prospective study. *J. Pediatr. Psychol.* 25 79–91. 10.1093/jpepsy/25.2.79 10820946

[B39] Smith S. K. Zimmerman S. Williams C. S. Preisser J. S. Clipp E. C. (2008). Post-traumatic stress outcomes in non-Hodgkin’s lymphoma survivors. *J. Clin. Oncol.* 26 934–941. 10.1200/JCO.2007.12.3414 18281667 PMC3025533

[B40] Stuber M. L. Meeske K. A. Krull K. R. Leisenring W. Stratton K. Kazak A. E. (2010). Prevalence and predictors of Posttraumatic Stress Disorder in adult survivors of childhood cancer: a report from the Childhood Cancer Survivor Study. *Pediatrics* 125 1124–1134. 10.1542/peds.2009-2308 PMC3098501 20435702

[B41] Stuber M. L. Meeske K. A. Leisenring W. Stratton K. Zeltzer L. K. Dawson K. (2011). Defining medical posttraumatic stress among young adult survivors in the Childhood Cancer Survivor Study. *Gen. Hosp. Psychiatry* 33 347–353. 10.1016/j.genhosppsych.2011.03.015 21762831 PMC3140002

[B42] Taïeb O. Moro M. R. Baubet T. Revah-Lévy A. Flament M. F. (2003). Posttraumatic stress symptoms after childhood cancer. *Eur. Child Adolesc. Psychiatry* 12 255–264. 10.1007/s00787-003-0352-0 14689257

[B43] Taylor N. Absolom K. Snowden J. Eiser C. Late Effects Group Sheffield (2012). Need for psychological follow-up among young adult survivors of childhood cancer. *Eur. J. Cancer Care (Engl.)* 21 52–58. 10.1111/j.1365-2354.2011.01281.x 21883564

[B44] Teall T. Barrera M. Barr R. Silva M. Greenberg M. (2013). Psychological resilience in adolescent and young adult survivors of lower extremity bone tumors. *Pediatr. Blood Cancer* 60 1223–1230. 10.1002/pbc.24441 23255460

[B45] Tremolada M. Bonichini S. Aloisio D. Schiavo S. Carli M. Pillon M. (2013). Post-traumatic stress symptoms among mothers of children with leukemia undergoing treatment: a longitudinal study. *Psychooncology* 22 1266–1272. 10.1002/pon.3132 22777982

[B46] Varela V. S. Ng A. Mauch P. Recklitis C. J. (2013). Posttraumatic stress disorder (PTSD) in survivors of Hodgkin’s lymphoma: prevalence of PTSD and partial PTSD compared with sibling controls. *Psychooncology* 22 434–440. 10.1002/pon.2109 22162210 PMC3908687

[B47] Yi J. Kim M. A. (2014). Postcancer experiences of childhood cancer survivors: how is posttraumatic stress related to posttraumatic growth? *J. Trauma. Stress* 27 461–467. 10.1002/jts.21941 25158639

[B48] Zebrack B. (2008). Information and service needs for young adult cancer patients. *Support Care Cancer* 16 1353–1360. 10.1007/s00520-008-0435-z 18386075

[B49] Zebrack B. J. Stuber M. L. Meeske K. A. Phipps S. Krull K. R. Liu Q. (2012). Perceived positive impact of cancer among long-term survivors of childhood cancer: a report from the Childhood Cancer Survivor Study. *Psychooncology* 21 630–639. 10.1002/pon.1959 21425388 PMC3697081

[B50] Zebrack B. J. Zeltzer L. K. (2003). Quality of life issues and cancer survivorship. *Curr. Probl. Cancer* 27 198–211. 10.1016/S0147-0272(03)00027-8 12855951

[B51] Zeltzer L. K. Lu Q. Leisenring W. Tsao J. C. Recklitis C. Armstrong G. (2009). Psychosocial outcomes and health-related quality of life in adult childhood cancer survivors: a report from the Childhood Cancer Survivor Study. *Cancer Epidemiol. Biomarkers Prev.* 17 435–446. 10.1158/1055-9965.EPI-07-2541 18268128

[B52] Zimet G. D. Powell S. S. Farley G. K. Werkman S. Berkoff K. A. (1990). Psychometric characteristics of the Multidimensional Scale of Perceived Social Support. *J. Pers. Assess.* 55 610–617. 10.1080/00223891.1990.9674095 2280326

